# Treatment of complicated urinary tract infection and acute pyelonephritis by short-course intravenous levofloxacin (750 mg/day) or conventional intravenous/oral levofloxacin (500 mg/day): prospective, open-label, randomized, controlled, multicenter, non-inferiority clinical trial

**DOI:** 10.1007/s11255-017-1507-0

**Published:** 2017-01-20

**Authors:** Hong Ren, Xiao Li, Zhao-Hui Ni, Jian-Ying Niu, Bin Cao, Jie Xu, Hong Cheng, Xiao-Wen Tu, Ai-Min Ren, Ying Hu, Chang-Ying Xing, Ying-Hong Liu, Yan-Feng Li, Jun Cen, Rong Zhou, Xu-Dong Xu, Xiao-Hui Qiu, Nan Chen

**Affiliations:** 1 Department of Nephrology, Rui Jin Hospital Shanghai Jiao Tong University School of Medicine, No. 197 Rui Jin Er Road, Shanghai, 200025 China; 2 Department of Nephrology, Ren Ji Hospital Shanghai Jiao Tong University School of Medicine, No. 1630 Dong Fang Road, Pu Dong New Area, Shanghai, 200127 China; 3 Department of Nephrology, The Fifth People’s Hospital of Shanghai, Fudan University, No. 801 He Qing Road, Min Hang District, Shanghai, 200240 China; 4 Department of Infectious Disease, Beijing Chao-Yang Hospital, No. 8 Gong Ti South Road, Chao Yang District, Beijing, 100020 China; 5 Department of Infectious Disease, Peking University Third Hospital, No. 49 Hua Yuan North Road, Hai Dian District, Beijing, 100191 China; 6 Department of Nephrology, Beijing Anzhen Hospital, Capital Medical University, No. 2 An Zhen Road, Chao Yang District, Beijing, 100029 China; 7 Department of Nephrology, Rocket Force General Hospital, No. 16 Xin Jie Kou Wai Street, Xi Cheng District, Beijing, 100088 China; 8 Department of Infectious Disease, Beijing Friendship Hospital, Capital Medical University, No. 95 Yong An Road, Xi Cheng District, Beijing, 100050 China; 9 Department of Nephrology, The Second Affiliated Hospital of Zhejiang University School of Medicine, No. 88 Jiefang Road, Hangzhou, 310009 Zhejiang Province China; 10 Department of Nephrology, Jiangsu Province Hospital, No. 300 Guangzhou Road, Nanjing, 210029 Zhejiang Province China; 11 Department of Nephrology, The Second Xiangya Hospital of Central South University, No. 139 Renmin Middle Road, Changsha, 410011 Hunan Province China; 12 Department of Urology Surgery, Daping Hospital, No. 10 Changjiang zhi Road, Yuzhong District, Chongqing, 400042 China; 13 Department of Nephrology, Shanghai Construction Group Hospital, No. 666 Zhongshan North Road, Hongkou District, Shanghai, 200083 China; 14 Department of Nephrology, Central Hospital of Yangpu District, No. 450 Tengyue Road, Yangpu District, Shanghai, 200090 China; 15 Department of Nephrology, Central Hospital of Minhang District, Shanghai, No. 170 Shensong Road, Minhang District, Shanghai, 201199 China; 16 Department of Nephrology, Ningbo Medical Treatment Center Lihuili Hospital, No. 57 Xingning Road, Jiang Dong District, Ningbo, 315040 Zhejiang Province China

**Keywords:** Levofloxacin, Complicated urinary tract infection (cUTI), Acute pyelonephritis (APN), Non-inferiority trial

## Abstract

**Objective:**

To compare the efficacy and safety of short-course intravenous levofloxacin (LVFX) 750 mg with a conventional intravenous/oral regimen of LVFX 500 mg in patients from China with complicated urinary tract infections (cUTIs) and acute pyelonephritis (APN).

**Methods:**

This was a prospective, open-label, randomized, controlled, multicenter, non-inferiority clinical trial. Patients with cUTI and APN were randomly assigned to a short-course therapy group (intravenous LVFX at750 mg/day for 5 days) or a conventional therapy group (intravenous/oral regimen of LVFX at 500 mg/day for 7–14 days). The clinical, laboratory, and microbiological results were evaluated for efficacy and safety.

**Results:**

The median dose of LVFX was 3555.4 mg in the short-course therapy group and 4874.2 mg in the conventional therapy group. Intention-to-treat analysis indicated the clinical effectiveness in the short-course therapy group (89.87%, 142/158) was non-inferior to that in the conventional therapy group (89.31%, 142/159). The microbiological effectiveness rates were also similar (short-course therapy: 89.55%, 60/67; conventional therapy: 86.30%, 63/73; *p* *>* 0.05). There were no significant differences in other parameters, including clinical and microbiological recurrence rates. The incidence of adverse effects and drug-related adverse effects were also similar for the short-course therapy group (21.95%, 36/164; 18.90%, 31/164) and the conventional therapy group (23.03%, 38/165; 15.76%, 26/165).

**Conclusion:**

Patients with cUTIs and APN who were given short-course LVFX therapy and conventional LVFX therapy had similar outcomes in clinical and microbiological efficacy, tolerance, and safety. The short-course therapy described here is a more convenient alternative to the conventional regimen with potential implication in anti-resistance and cost saving.

**Electronic supplementary material:**

The online version of this article (doi:10.1007/s11255-017-1507-0) contains supplementary material, which is available to authorized users.

## Introduction

Urinary tract infection (UTI) is one of the most common bacterial infections and is particularly common in women [1]. Complicated UTIs (cUTIs) and acute pyelonephritis (APN), a subset of cUTI, are treated by management of the underlying functional or structural abnormality, administration of appropriate antibiotics, and symptom management with or without hospitalization [2]. cUTIs can lead to bacteremia and are associated with a high mortality rate. Prolonged or repeated administration of antibiotics is required for the treatment of cUTIs, but this can lead to the development of antibiotic resistance. Extended spectrum β-lactamase (ESBL)-producing *Escherichia coli* is the most common pathogen responsible for cUTIs, but many other Gram-negative and Gram-positive species have been isolated from patients [3], and the prevalence of different pathogens depends on patient sex and the presence of uncomplicated UTI or cUTI. Quinolones are the drug of choice for treatment of cUTIs, but *E. coli* has a ciprofloxacin resistance rate as high as 58.3% in China [4]. There is currently no consensus on the optimal therapeutic regimen for the treatment of cUTIs while preventing the development of drug resistance.

Levofloxacin (LVFX) is a quinolone that is widely used to treat cUTIs and APN [5]. There are several therapeutic regimens that employ LVFX for treatment of these infections. A study of patients with APN indicated that a high-dose and short-term LVFX regimen (750 mg/day for 5 days) was non-inferior to a standard ciprofloxacin regimen (twice daily for 10 days) [6]. The USA has approved a high-dose and short-term LVFX regimen for the treatment of cUTIs, APN, and other infectious diseases [7]. Pharmacokinetic and pharmacodynamic studies of LVFX have confirmed that its therapeutic efficacy depends on the dose and the ratio of the area under the time–concentration curve to the minimum inhibitory concentration (AUC/MIC) [8]. This is considered a key pharmacodynamic parameter that determines the optimal bactericidal activity and prevents the development of resistance. There is also evidence that the increased ratio of peak plasma concentration of LVFX to MIC (Cmax/MIC) can prevent the development of resistance [9–11]. Other research showed that an oral regimen of LVFX at 750 mg per day doubles the serum AUC and Cmax relative to an oral regimen of LVFX at 500 mg per day [12, 13].

The duration of LVFX therapy is important for improving efficacy and reducing the development of resistance. Thus, short-term therapy with LVFX at a high dose (750 mg/day for 5 days) may be preferable to a more prolonged treatment with a lower dose [6, 14]. In addition, a short-term and high-dose LVFX regimen may require fewer medical resources and improve patient outcomes. However, limited data on this regimen are available for patients in China.

This study compared the efficacy and safety of intravenous LVFX at 750 mg per day for 5 days with an intravenous/oral regimen of LVFX at 500 mg per day for 7–14 days in the treatment of patients with cUTIs and APNs.

## Materials and methods

### Study design

This was a prospective, open-label, controlled, multicenter study that recruited patients from 16 clinical centers between October 2012 and July 2014. This trial was conducted according to the Helsinki guidelines and the guidelines for Chinese Good Clinical Practice (GCP). All patients provided informed consent for participation.

### Study population

Study subjects were male or female patients who were at least 18 years old, were inpatients (*n* = 196) or outpatients (*n* = 121), had diagnoses of cUTI or APN (females only) [8], and were willing to participate in this study and cooperate with clinicians, based on the provision of informed consent. Patients who failed after 72 h therapy with other non-quinolone antibiotics were eligible as well.

Patients were excluded if they were pregnant, breast-feeding, or preparing for pregnancy during study period; received other systemic antimicrobial therapy due to a UTI; required or received a long-lasting indwelling catheter; had complete urinary tract obstruction; had urinary tract tumors; received urinary tract surgery or lithotripsy (due to renal calculus) in the 7 days before study onset; had a history of epilepsy; had a history of quinolone-induced tendon lesions; had a history of prolonged QT interval and/or prolonged QT interval on recruitment; were allergic to levofloxacin or other quinolones; received any antibiotic therapy within 72 h before study onset and if their condition improved within 72 h before study onset; had severe heart disease, liver disease (≥2 times upper limit of normal liver enzymes), or pre-existing kidney disease (creatinine clearance <50 mL/min), or if the investigators judged the patient ineligible; received therapy with at least one drug used in this study in the 4 weeks before study onset; had at least one health-threatening clinical disease or abnormality that could affect the quality of data.

Patients were allowed to exit the study if they wanted to withdraw; were lost to follow-up; did not achieve remission after 72 h of therapy (although these data were included in the final analysis); received therapy with other drugs that were not allowed; became pregnant; had any pathological event, clinical adverse event, or a physical condition that made the clinicians think continued participation was incompatible with the best interest of patient.

### Drug regimens

Patients were randomly assigned to 2 groups: a LVFX 750 mg (5 days) group and a LVFX 500 mg (7–14 days) group. Patients in the LVFX 750-mg group received intravenous infusion of LVFX (750 mg/150 mL) once daily for 5 consecutive days. Patients in the LVFX 500-mg group received intravenous infusion of LVFX (500 mg/100 mL) once daily and were then shifted to an oral regimen of LVFX (500 mg/tablet) once daily for 7–14 days. The shift from an intravenous to an oral regimen of LVFX was determined according to the mitigation of clinical symptoms (fever and other symptoms). Drugs were purchased from Daiichi Sankyo.

### Evaluation of therapeutic efficacy

The clinical effectiveness rate at the end of therapy (EOT) was the major measure of therapeutic efficacy (day 6 + 1 in the LVFX 750-mg group and day 8–15 in the LVFX 500-mg group). The clinical efficacy was classified as complete remission, remission, non-remission, and not applicable (NA). The effectiveness rate was calculated based on complete remission and remission. Complete remission was defined as the complete absence of clinical symptoms and signs without further antibiotic therapy. Remission was defined as the significant reduction in clinical symptoms and signs with a requirement for further antibiotic therapy. Non-remission was defined as the deterioration or recurrence of clinical symptoms/signs with a requirement for further antibiotic therapy. NA was defined as a loss to follow-up within 3 days after therapy or use of other antibiotic(s) for reasons other than a UTI.

Several secondary parameters were used to evaluate therapeutic efficacy. These were: clinical effectiveness rate at the EOT and at the second and third hospital visits; microbiological effectiveness rate at the EOT based on clearance/suspected clearance, continuance/suspected continuance, recurrence, replacement, new infection or NA; body temperature (BT) at the EOT, and at the second and third hospital visits (compared to baseline); time to remission of clinical symptoms/signs; white blood cell count (WBC), C-reactive protein (CRP) level, and erythrocyte sedimentation rate (ESR) at the EOT, and at the second and third hospital visits (compared to baseline); recurrence rate during the follow-up period (9–15 days after the EOT), including clinical recurrence rate and microbiological recurrence rate. Clinical recurrence refers to the recurrence of clinical symptoms and signs. Microbiological recurrence refers to positive results (≥10^4^ cfu/mL) in a urine culture at any hospital visit after confirmation of bacterial clearance by urine culture. Patients with clearance/suspected clearance, replacement, or new infection were included for the calculation of the microbiological effectiveness rate.

Safety was evaluated based on abnormal laboratory parameters, adverse events, and severe adverse events. The correlation between adverse events and antibiotic therapy was evaluated and classified as definite, possible, probable, probably not, possibly not, and NA. Definite, possible, probable, and NA adverse events were regarded as adverse events of antibiotic therapy. The influence of antibiotic therapy on vital signs and electrocardiogram results was also evaluated.

### Analysis sets

The intention-to-treat (ITT) set included patients who received at least one of the above-mentioned therapies and had available data. For patients who were not observed during the whole study period, the last observation carried forward (LOCF) method was employed. The bacteriological ITT (B-ITT) set included patients who were positive in the baseline microbiological examination and had a detectable pathogen.

The per-protocol (PP) set included patients who did not violate the study protocol. These patients received therapy for at least 3 days (second hospital visit) and had data available for evaluation of therapeutic efficacy. The bacteriological PP (B-PP) set included patients who were positive in the baseline microbiological examination and had a detectable pathogen.

Safety set (SS) included patients who received therapy at least once.

### Sample size estimation

A review of the literature led to an estimated response rate of 76–96% for the LVFX 750 mg regimen and 74–94% for the LVFX 500 mg regimen. The non-inferiority margin for acute pyelonephritis (APN) treatment is 15%. The supplemental tables show the sample size per group under power 90 or 80% for a one-sided significance level of 2.5% (Tables S1 and S2).

### Statistical analysis

This was a non-inferiority clinical trial that compared two LVFX regimens for treatment of cUTI and APN. The non-inferiority margin was −15%. Confidence intervals (CIs) were estimated to determine the success rates of the 2 groups. The primary and secondary endpoints were clinical success rate and microbiologic eradication rate, respectively. The Cochran–Mantel–Haenszel (CMH) test was used to assess correlations between the different treatment groups and among different strains of microbes. For comparison of baseline variables, continuous variables with normal distributions are presented as means and standard deviations, and an independent samples *t* test was used to compare the groups. Continuous variables with skewed distributions are presented as medians and inter-quartile ranges (IQRs), and the Mann–Whitney *U* test was used to compare the groups. Categorical baseline variables and adverse events are presented as counts and percentages and compared by a Chi-square test or Fisher’s exact test. Statistical analyses were performed with IBM SPSS statistical software version 22 for Windows (IBM Corp., Armond, New York, USA). A 2-tailed *p* value below 0.05 was considered statistically significant.

## Results

### Baseline characteristics of study subjects

**Fig. 1:**
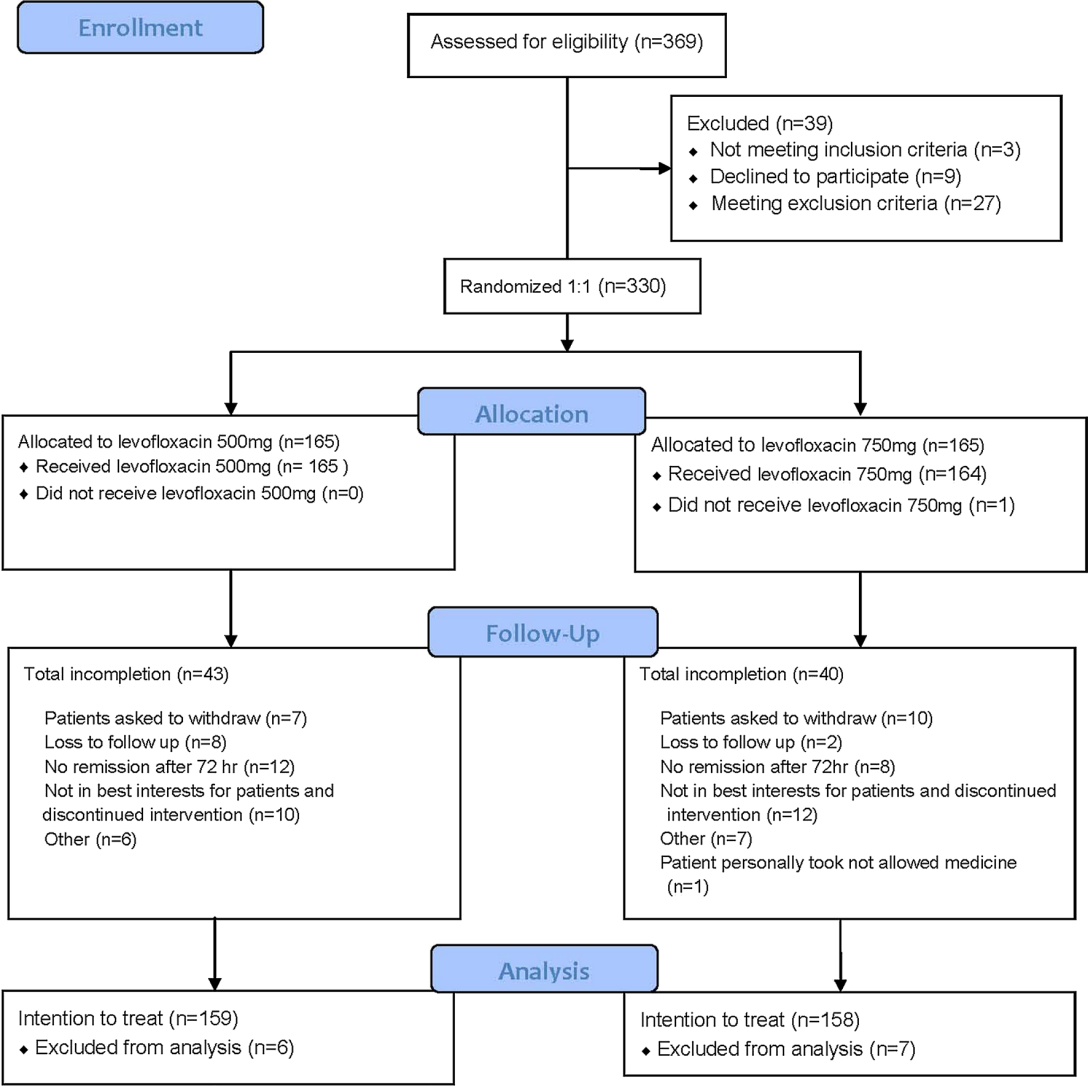
Flow diagram

We assessed 369 patients for eligibility. Based on the inclusion and exclusion criteria and patients’ willingness to participate, we enrolled 330 subjects, with 165 in the LVFX 500-mg group and 165 in the LVFX 750-mg group. A total of 122 subjects in LVFX 500-mg group and 125 in LVFX 750-mg group completed the therapeutic regimens. A total of 83 subjects did not complete the study because continuation was incompatible with their best interests, remission did not occur after 72 h of therapy, they had a desire to withdraw, they were lost to follow-up, they received therapy with drugs that were not allowed, or other reasons (Table 1 and flow diagram Fig. 1).

**Table 1:** Disposition of patients with cUTI or APN who were given the LVFX 500-mg regimen and the LVFX 750-mg regimen

	LVFX 500 mg	LVFX 750 mg	Total
Number of patients	165 (100.00%)	165 (100.00%)	330 (100.00%)
Completed protocol	122 (73.94%)	125 (75.76%)	247 (74.85%)
Withdrew	43 (26.06%)	40 (24.24%)	83 (25.15%)
Not in best interests^*^	10 (6.06%)	12 (7.27%)	22 (6.67%)
No remission after 72 h	12 (7.27%)	8 (4.85%)	20 (6.06%)
Withdrawal	7 (4.24%)	10 (6.06%)	17 (5.15%)
Loss to follow-up	8 (4.85%)	2 (1.21%)	10 (3.03%)
Others	6 (3.64%)	8 (4.85%)	13 (3.94%)

* Any pathological event, clinical adverse event, or abnormal physical condition that made investigators consider continued participation was incompatible with the best interests of the patient

The baseline characteristics of the LVFX 500 mg and LVFX 750-mg groups, including age, gender, height, weight, history of allergies, and diagnostic results, were similar (*p* > 0.05 for all comparisons, Table 2). In addition, there were 96 inpatients and 63 outpatients in the LVFX 500-mg group, and 86 inpatients and 58 outpatients in the LVFX 750-mg group (*p* = 0.593). The median treatment time (*p* < 0.001) and median exposure dose (*p* < 0.001) were significantly greater in LVFX 500-mg group. Table S3 shows the results of urine cultures (performed before initiation of therapy) in the ITT population.

**Table 2:** Baseline characteristic of patients with cUTIs and APN who were given the LVFX 500 mg regimen and the LVFX 750 mg regimen

Variable	LVFX 500 mg	LVFX 750 mg	*p* value
	(*N* = 159)	(*N* = 158)	
Age (years), mean ± SD	50.18 ± 17.42	49.08 ± 17.37	0.574
Sex			0.512
Male	22 (13.84%)	18 (11.39%)	
Female	137 (86.16%)	140 (88.61%)	
Height (cm), mean ± SD	160.78 ± 6.08	161.17 ± 6.07	0.572
Weight (kg), mean ± SD	59.68 ± 9.74	60.65 ± 10.76	0.404
History of allergies	20 (12.58%)	16 (10.13%)	0.491
Diagnostic result			0.697
cUTI	90 (56.6%)	86 (54.43%)	
APN	69 (43.4%)	72 (45.57%)	
Source			0.593
Inpatients	96 (60.38%)	100 (63.29%)	
Outpatients	63 (39.62%)	58 (36.71%)	
Treatment time (days), median (IQR)	9 (7, 13)	5 (5, 5)	<0.001
Exposure dose (mg), median (IQR)	4500 (3500, 6500)	3750 (3750, 3750)	<0.001

### Clinical success rate

ITT analysis indicated the clinical success rate was 89.31% in the LVFX 500-mg group and 89.87% in the LVFX 750-mg group. The 95% CI of the differences between these groups was −6.16% to 7.29%, significantly higher than the non-inferiority margin of −15% (*p* < 0.05). PP analysis indicated the clinical success rate was 90.34% in LVFX 500-mg group and 93.10% in LVFX 750-mg group. The 95% CI of the differences between these groups was −3.58 to 9.09%, also significantly higher than the non-inferiority margin of −15% (*p* < 0.05). Analysis of the clinical success rate indicated that the LVFX 500-mg group was not inferior to the LVFX 750-mg group. The clinical success rates were significantly better for APN than for cUTI in both the LVFX 500-mg group and the LVFX 750-mg group (*p* < 0.05 for both comparisons) (Table 3).

**Table 3:** Clinical success rate based on intention-to-treat analysis and per-protocol analysis

Clinical success rate*	ITT		PPS	
	LVFX 500 mg^#^	LVFX 750 mg^#^	LVFX 500 mg	LVFX 750 mg
	*N* = 159	*N* = 158	*N* = 145	*N* = 145
APN	**95.65% (66/69)**	95.83% (69/72)		
cUTI	**84.44% (76/90)**	84.88% (73/86)		
Clinical success rate	89.31% (142/159)	89.87% (142/158)	90.34% (131/145)	93.10% (135/145)
Difference of clinical success (95% CI)	0.57 (−6.16,7.29)		2.76 (−3.58, 9.09)	

The *P* value was < 0.05 (0.0234) for the difference between APN and cUTI of LVFX 500 mg

* Including patients who achieved complete success and remission

^**#**^ *p* < 0.05, significantly different for patients with APN and cUTI

### Microbiologic eradication rate

ITT analysis indicated the microbiologic eradication rate was 86.30% in the LVFX 500-mg group and 89.55% in the LVFX 750-mg group (*p* > 0.05). The microbiologic eradication rate was significantly higher for APN than for cUTI in the LVFX 500-mg group (100 versus 72.97%, *p* = 0.003), but not in the LVFX 750-mg group (*p* > 0.05) (Table 4).

**Table 4:** Microbiologic eradication rates of APN and cUTI in patients given the LVFX 500 mg regimen and the LVFX 750 mg regimen

Diagnosis	LVFX 500 mg^*^	LVFX 750 mg	*p* value
APN	100.00% (36/36)	91.67% (33/36)	
cUTI	72.97% (27/37)	87.10% (27/31)	
Effectiveness	86.30% (63/73)	89.55% (60/67)	0.556

*p < 0.05, significantly better effectiveness against APN than cUTI

### Clinical success time and time after last treatment

**Fig. 2:**
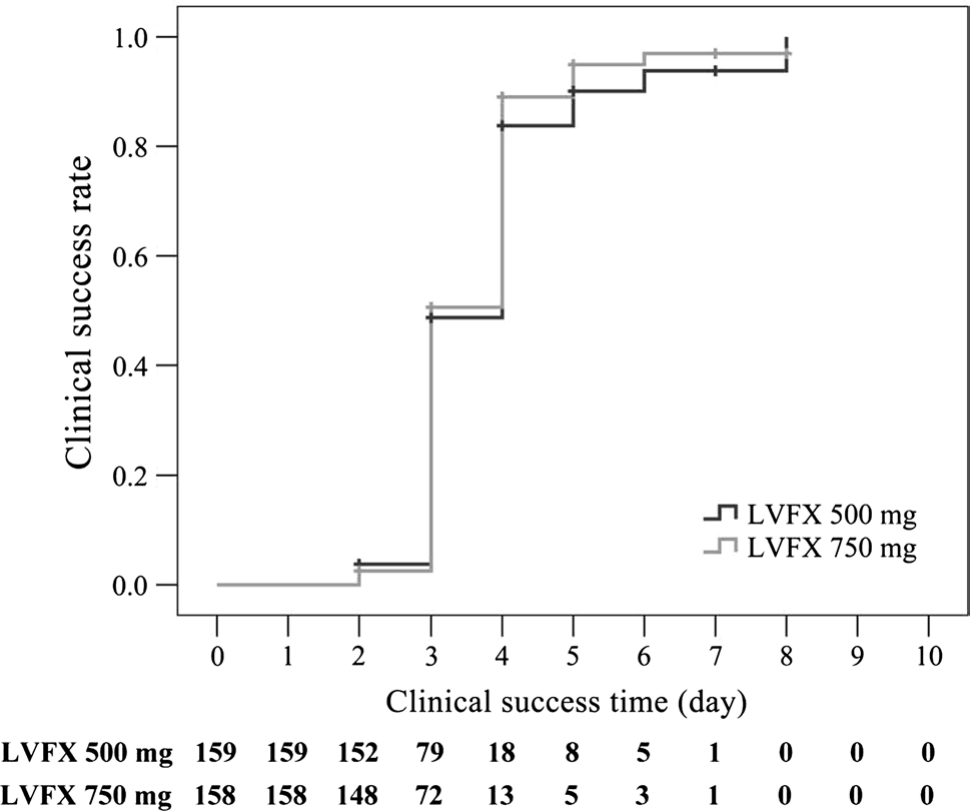
Time course of clinical success for patients in the LVFX 500-mg group and the LVFX 750-mg group^*^

**Fig. 3:**
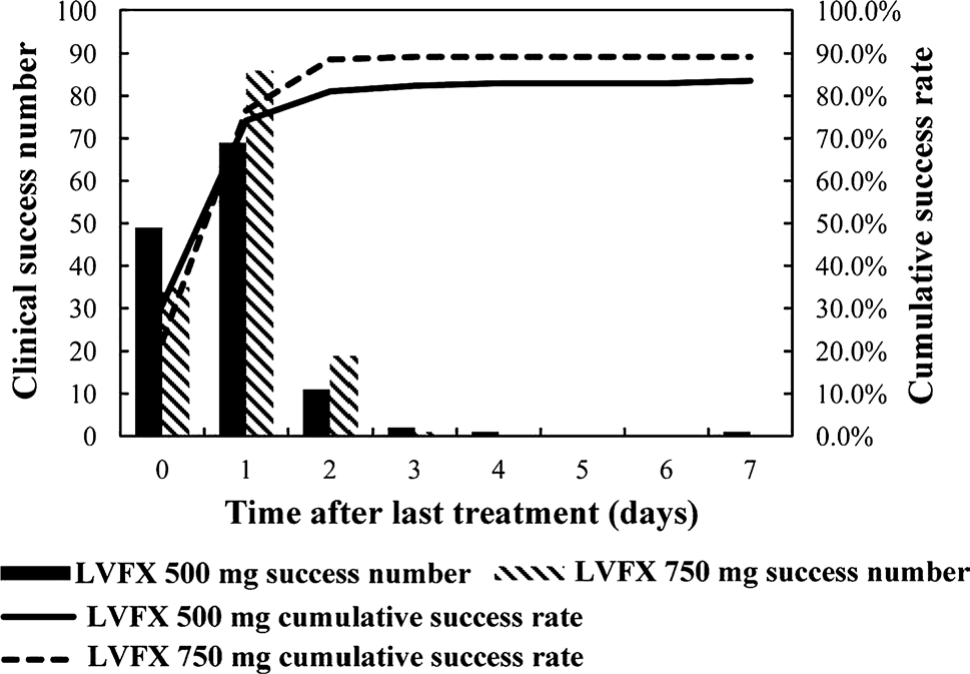
Time course of clinical success after the last treatment for patients in the LVFX 500-mg group and the LVFX 750-mg group

The median clinical success time was 4 days in the LVFX 500-mg group and 3 days in the LVFX 750-mg group (*p* > 0.05) (Fig. 2). Moreover, efficacy improved after cessation of treatment, especially in the LVFX 750-mg group (Fig. 3).

### Safety assessment

The total adverse event rate was 23.03% in the LVFX 500-mg group and 21.95% in the LVFX 750-mg group (*p* > 0.05) (Table 5). Analysis of all adverse events in LVFX 500-mg group indicated that 15.76% were associated with the study drug, 1.21% were severe adverse events, and 6.06% were associated with withdrawal from the study. Analysis of all adverse events in LVFX 750-mg group indicated that 18.90% were associated with the study drug, 0.61% were severe adverse events, and 6.71% were associated with withdrawal from the study. The two groups had no significant differences in any individual adverse events or examination results (*p* > 0.05 for all comparisons) (Table 5).

**Table 5:** Adverse events among patients in the LVFX 500-mg group and the LVFX 750-mg group

	LVFX 500 mg		LVFX 750 mg		*p* value
	(*N* = 165)		(*N* = 164)		
	*N* (incidence)	*N* (case-time)	*N* (incidence)	*N* (case-time)	
Total	38 (23.03%)	52	36 (21.95%)	57	0.792
Related to drugs*	26 (15.76%)	36	31 (18.90%)	46	0.071
Severe	2 (1.21%)	2	1 (0.61%)	1	1.000
Resulting in loss to follow-up*	10 (6.06%)	10	11 (6.71%)	15	0.686
Examinations	16 (9.70%)	17	14 (8.54%)	17	0.702
Reduction in leukocyte count	9 (5.45%)	9	9 (5.49%)	9	1.000
Reduction in neutrophil count	2 (1.21%)	2	3 (1.83%)	3	1.000
Increased ALT	2 (1.21%)	2	2 (1.22%)	2	1.000
Increased ASP	2 (1.21%)	2	3 (1.83%)	2	1.000
Increased platelet count	2 (1.21%)	2	0 (0%)	0	0.489
Increased blood pressure	0 (0%)	0	1 (0.61%)	1	1.000
Gastrointestinal	4 (2.42%)	6	7 (4.27%)	10	0.358
Reaction at injection site	7 (4.24%)	9	3 (1.83%)	4	0.199
Cutaneous/subcutaneous	3 (1.82%)	2	3 (1.83%)	3	1.000
Nervous system/mental	1 (0.61%)	0	4 (2.44%)	7	0.371
Immune	1 (0.61%)	1	1 (0.61%)	1	1.000
Infection	7 (4.24%)	1	1 (0.61%)	1	0.067
Hepatobiliary	0 (0%)	0	1 (0.61%)	1	1.000
Metabolic/nutritional	1 (0.61%)	0	3 (1.83%)	1	0.623
Musculoskeletal/connective tissue	0 (0%)	0	1 (0.61%)	1	1.000

* The correlation between adverse events and drugs was classified as definite, probable, or possible

## Discussion

The present study compared the safety and efficacy of a short-term and high-dose LVFX regimen (750 mg/day for 5 days) with a routine LVFX regimen (500 mg/day plus oral regimen of LVFX for 7–14 days) for the treatment of patients with cUTI or APN. This study is the first and largest of its kind for the Chinese population to examine this short-term and high-dose treatment regimen for the most common type of bacterial infection [1]. The results showed that these two therapies had similar clinical efficacy, microbiological efficacy, and tolerance. These results indicate that the short-term and high-dose LVFX therapy that is approved in China and the USA [14] is also suitable for patients in Asia with cUTIs or APN. The results of the present study also indicate that the therapeutic effectiveness of each regimen continued for at least 7 days after discontinuation and that this effect was more obvious for the LVFX 750-mg regimen. Taken together, these results support the efficacy and safety of the LVFX 750-mg regimen.

Another important finding of the present study is that the total duration of the LVFX 750 mg regimen was 50% shorter and the total dose of the LVFX 750 mg regimen was 27% less. The present study did not compare the costs of the different LVFX regimens, but these data strongly suggest that the LVFX 750 mg regimen is associated with reduced need for medical resources and reduced costs. These factors are particularly noteworthy for developing countries with large populations and more limited medical resources.

There is a very high incidence of quinolone-resistant *E. coli* in China, and the MIC_90_ of quinolones is as high as 16 mg/L for this species [15]. Clinical protocols for treatment of UTIs, especially cUTIs, must be effective against *E. coli* because this species is the most common causative agent [16]. The development of new antibiotics has slowed, so it is necessary to optimize the regimens of available antibiotics to treat UTI and APN. LVFX is a dose-dependent antibiotic that is excreted in the urine. High-dose LVFX leads to increased concentration in the urine, prolonged (8–12 h) activity against *E. coli*, and efficacy against *E. coli* with MICs up to 32 µg/mL [17]. Although the present study did not measure the sensitivity of isolated pathogens to LVFX, the bacterial clearance rate in the LVFX 750-mg group was higher than in the LVFX 500-mg group among patients with cUTIs. This indicates that the LVFX 750 mg regimen may achieve better outcomes in cUTI patients.

A 2008 study in the USA compared a LVFX regimen (750 mg per day for 5 days) with a ciprofloxacin regimen (400/500 mg twice daily for 10 days) for treatment of cUTIs and APN [14]. The results showed that these regimens had comparable effectiveness and safety. However, the ciprofloxacin regimen described in this previous study is probably not suitable for the treatment of cUTI and APN in China, where the ciprofloxacin resistance rate for *E. coli* can exceed 50% [4]. A recent study compared a ceftolozane–tazobactam regimen with a levofloxacin regimen (750 mg/day for 5 days) for treatment of cUTIs and APN [18]. The results indicated that the ceftolozane–tazobactam regimen was better than the high-dose levofloxacin regimen. Although this study enrolled 1083 patients from 209 sites throughout the world, none of the patients were from China. Thus, it remains to be determined whether this ceftolozane–tazobactam regimen is also more effective in Chinese patients with cUTIs or APN.

In conclusion, Chinese patients with cUTIs and APN who were given intravenous LVFX at 750 mg per day for 5 days or an intravenous/oral regimen of LVFX at 500 mg per day for 7–14 days had similar outcomes in terms of clinical and microbiological efficacy, tolerance, and safety. The LVFX 750 mg regimen may be preferred for the treatment of these infections because its duration was 50% less and the total drug dose was 23% less.

## Electronic supplementary material

Below is the link to the electronic supplementary material.

Supplementary material 1 (DOCX 25 kb)
