# Supplementary material for: Treatment of complicated urinary tract infection and acute pyelonephritis by short-course intravenous levofloxacin (750 mg/day) or conventional intravenous/oral levofloxacin (500 mg/day): prospective, open-label, randomized, controlled, multicenter, non-inferiority clinical trial
Source: Int Urol Nephrol. 2017 Jan 20;49(3):499–507. doi: 10.1007/s11255-017-1507-0 (PMC5321781; doi:10.1007/s11255-017-1507-0)
Supplement: Supplementary file 1 — Supplementary material 1 (DOCX 25 kb) [file 11255_2017_1507_MOESM1_ESM.docx]

**Supplemental Table 1.** Sample size per group for a power 80%.

| **Sample size**  **per group** | | **Response rate (RR) in 750-mg gp (%)** | | | | | | | | | | |
| --- | --- | --- | --- | --- | --- | --- | --- | --- | --- | --- | --- | --- |
|  |  | **76** | **78** | **80** | **82** | **84** | **86** | **88** | **90** | **92** | **94** | **96** |
| **RR in 500-mg gp (%)** | **94** | - | - | - | - | - | - | - | - | - | 40 | 26 |
|  | **92** | - | - | - | - | - | - | - | - | 52 | 36 | 25 |
|  | **90** | - | - | - | - | - | - | - | 63 | 45 | 32 | 24 |
|  | **88** | - | - | - | - | - | - | 74 | 54 | 40 | 30 | 22 |
|  | **86** | - | - | - | - | - | 85 | 62 | 46 | 35 | 27 | 21 |
|  | **84** | - | - | - | - | 94 | 70 | 53 | 41 | 32 | 25 | 20 |
|  | **82** | - | - | - | 103 | 77 | 59 | 46 | 36 | 29 | 23 | 18 |
|  | **80** | - | - | 112 | 84 | 65 | 51 | 40 | 32 | 26 | 21 | 17 |
|  | **78** | - | 120 | 91 | 70 | 55 | 44 | 36 | 29 | 24 | 20 | 16 |
|  | **76** | 128 | 97 | 75 | 59 | 48 | 39 | 32 | 27 | 22 | 19 | 16 |
|  | **74** | 102 | 80 | 63 | 51 | 42 | 35 | 29 | 24 | 20 | 17 | 15 |

**Supplemental Table 2.** Sample size per group for a power of 90%.

| **Sample size**  **per group** | | **Response rate (RR) in 750-mg gp (%)** | | | | | | | | | | |
| --- | --- | --- | --- | --- | --- | --- | --- | --- | --- | --- | --- | --- |
|  |  | **76** | **78** | **80** | **82** | **84** | **86** | **88** | **90** | **92** | **94** | **96** |
| **RR in 500-mg gp (%)** | **94** | - | - | - | - | - | - | - | - | - | 53 | 35 |
|  | **92** | - | - | - | - | - | - | - | - | 69 | 48 | 33 |
|  | **90** | - | - | - | - | - | - | - | 85 | 60 | 43 | 31 |
|  | **88** | - | - | - | - | - | - | 99 | 72 | 53 | 39 | 29 |
|  | **86** | - | - | - | - | - | 113 | 83 | 62 | 47 | 36 | 28 |
|  | **84** | - | - | - | - | 126 | 93 | 70 | 54 | 42 | 33 | 26 |
|  | **82** | - | - | - | 138 | 103 | 79 | 61 | 48 | 38 | 31 | 24 |
|  | **80** | - | - | 150 | 112 | 86 | 68 | 54 | 43 | 35 | 28 | 23 |
|  | **78** | - | 161 | **121** | 94 | 74 | 59 | 48 | 39 | 32 | 26 | 22 |
|  | **76** | 171 | 129 | 100 | 79 | 64 | 52 | 43 | 24 | 29 | 24 | 20 |
|  | **74** | 137 | 107 | 85 | 68 | 56 | 46 | 38 | 32 | 27 | 23 | 19 |

###### Supplemental Table 3. Results of urine cultures in the intention to treat population. Some patients had more than one pathogen, and others had no detectable pathogens.

| **Species** | **LVFX 500-mg group (N=159)** | **LVFX 750-mg group (N=158)** |
| --- | --- | --- |
| *Escherichia coli* | 64 | 53 |
| *Enterococcus faecalis* | 2 | 4 |
| *Klebsiella pneumoniae* | 3 | 2 |
| *Streptococcus agalactiae* | 1 | 3 |
| *Staphylococcus epidermidis* | 1 | 0 |
| *Enterobacter aerogenes* | 0 | 1 |
| *Citrobacter freundii* | 1 | 0 |
| *Staphylococcus saprophyticus* | 1 | 1 |
| *Streptococcus* | 0 | 1 |
| *Acinetobacter lwoffi* | 1 | 0 |
| *Gemella morbillorum* | 1 | 0 |
| *Enterococcus durans* | 1 | 0 |
| *Balcillus proteus mirabilis* | 1 | 1 |
| *Staphylococcus haemolyticus* | 1 | 1 |
| *Enterobacter cloacae* | 0 | 1 |
